# Characteristics of peritumoral pseudocapsule in small renal cell carcinoma and its influencing factors

**DOI:** 10.1002/cam4.4991

**Published:** 2022-06-29

**Authors:** Xiang Dong, Sheng Pan, Xiaodie Zhou, Wenliang Ma, Hongqian Guo, Weidong Gan

**Affiliations:** ^1^ Department of Urology, Affiliated Drum Tower Hospital Medical School of Nanjing University Nanjing China; ^2^ Department of Urology, Nanjing Drum Tower Hospital Clinical College of Traditional Chinese and Western Medicine Nanjing University of Chinese Medicine Nanjing China; ^3^ Department of Pathology, Nanjing Jinling Hospital Nanjing University School of Medicine Nanjing China

**Keywords:** CT, enucleation, pseudocapsule, renal cell carcinoma

## Abstract

**Background:**

The aim of this study was to investigate the peritumoral pseudocapsule (PC) status and identify the factors influencing PC status in small renal cell carcinoma (RCCs).

**Methods:**

A total of 147 patients with small RCC (≤4 cm) who had undergone tumor enucleation (TE) were assigned into three groups according to PC status: complete PC, PC absence, and PC invasion. Computed tomography (CT) imaging and clinicopathological features were compared among the three groups. Univariate and multivariate analyses were performed to identify factors associated with incomplete PC.

**Results:**

The number of patients with complete PC, PC absence, and PC invasion was 87 (59%), 20 (14%), and 40 (27%), respectively. Compared with the other two groups, tumors with complete PC were most common in clear cell RCC (CCRCC) and showed a hyperenhancement pattern (92%) and clear boundary (63%) on CT scanning images (*p* < 0.001). PC absence was most common in female patients (50%), whereas PC invasion was more common in male patients (85%) (*p* = 0.017). The tumor diameter in the PC absence group (2.24 ± 0.93 cm) was shorter compared with that of the complete PC group (2.88 ± 0.76 cm) and PC invasion group (3.16 ± 0.64 cm) (*p* < 0.001). Univariate and multivariate analysis showed that hypoenhancement pattern, unclear boundary, and non‐CCRCC subtype were independent risk factors of incomplete PC.

**Conclusions:**

Hypoenhancement pattern, unclear boundary, and non‐CCRCC subtype were significant predictors of incomplete PC in small RCCs. It remains to be established whether TE is an appropriate procedure for patients with incomplete PC.

## Graphical abstract

The aim of this study was to investigate the pseudocapsule status and identify the factors influencing PC status in small renal cell carcinomas. Hypoenhancement pattern, unclear boundary, and non‐CCRCC subtype were independent risk factors of incomplete PC.

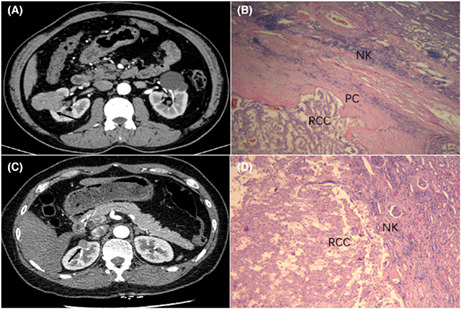

## 1 INTRODUCTION

Currently, renal cell carcinoma (RCC) is the most common malignant renal tumor, accounting for 90% of all tumor diagnoses.^1^ Advancement in imaging diagnostic technology has resulted in increased diagnosis of patients with small RCCs (maximum axial diameter ≤4 cm) at an early stage.^2^ Nephron‐sparing surgery (NSS) is recommended as the standard procedure for patients with small RCCs.^1^, ^3^ Standard partial nephrectomy (PN) is a type of NSS that involves removal of the entire tumor along with partial surrounding renal parenchyma to obtain a negative surgical margin.^4^ However, this procedure may increase the risk of intraoperative bleeding and postoperative renal function impairment. Thus, there is a need to develop a new and effective surgical procedure for patients with small RCCs. Recently, tumor enucleation (TE) surgery has been developed as an effective surgical procedure for small RCCs. This procedure involved excision of the tumor along a natural surgical dissection plane between the normal renal parenchyma and peritumoral pseudocapsule (PC).^1^, ^4^ The oncological outcomes of TE are comparable to those of PN, but with less renal parenchyma injury and intraoperative blood loss.

Peritumoral PC is the histological basis for TE surgery in RCC. It is composed of dense fibrous connective tissue that limits tumor cells infiltration into the normal surrounding renal parenchyma.^5^, ^6^ It has been shown that PC status is associated with the choice of surgical procedure and the prognosis of patient with renal tumors. For patients with complete PC, TE results in a low rate of positive surgical margin, and the opposite is true for patients with incomplete PC. Compared with patients with complete PC, patients with PC invasion and absence may have a worse prognosis.^4^, ^7^, ^8^, ^9^ Thus, accurate assessment of PC status is particularly important for patients with small RCC. Most previous studies investigating the PC status were focused on renal tumors.^3^, ^5^, ^6^, ^10^, ^11^, ^12^ Only a few studies have explored the PC status in localized RCCs.^7^, ^9^ To date, no study has reported the PC status in small RCCs after TE. The present study aimed to investigate the PC status and identify the factors influencing PC status in small RCCs.

## 2 METHODS

A total of 214 patients with small RCCs who had undergone TE in Nanjing Drum Tower Hospital were analyzed from January 2020 to January 2022. The inclusion criteria were as follows: (1) Patients with 18 or over 18 years old; (2) Pathologically confirmed RCCs; (3) Underwent preoperative contrast‐enhanced computed tomography (CT) scan; (4) Complete clinicopathological data. Exclusion criteria were as follows: (1) Rare subtype of RCCs; (2) Patients with lymph nodes or distant metastasis; (3) Multiple ipsilateral or bilateral RCCs; (4) Patients who received immune or targeted therapy before surgery. Finally, 147 patients were included in the study.

The following data were analyzed during the study: age, gender, body mass index (BMI), side, hypertension, smoking history, clinical symptom, tumor diameter, CT, and pathological findings. CT imaging features included necrosis/cystic components, homogeneity, enhancement pattern, and boundary. Heterogeneous and homogeneous enhancement were defined as the presence or absence of unenhanced areas in the tumor lesion, respectively.^13^ The definition of hyperenhancement and hypoenhancement was based on the degree of tumor lesion enhancement compared with the surrounding renal cortex on corticomedullary phase. We defined a clear boundary as the regular and distinct low‐attenuation rim at the tumor‐parenchyma interface on the corticomedullary phase images, based on reports from previous studies.^10^, ^14^, ^15^ If the rim was blurred or discontinuous, it was considered an unclear boundary. Pathological diagnosis was performed based on the 2016 World Health Organization classification. Small RCC was divided into low grade (Grade 1 and 2) and high grade (Grade 3 and 4) according to the 2016 World Health Organization/International Society of Urological Pathology (WHO/ISUP) nuclear grading system. But the system was not applicable for chromophobe RCC.^16^

Peritumoral PC status were evaluated by two independent observers and reviewed by an experienced uropathologist who was blinded to other clinical characteristics. To ensure accuracy and consistency, the evaluation of PC status was performed on the tumor‐parenchyma interface. Three or more slides with tumors surrounded by PC or renal parenchyma were considered to be a sufficient histologic material. The exception occurred when the size of the tumor was less than 2 cm. We classified PC status as complete PC, PC absence, and PC invasion. We defined complete PC as continuous PC without tumor infiltration in all slides, PC absence as the invisible PC with tumor cells immediately adjacent to the normal renal parenchyma in all slides, and PC invasion as tumor cells infiltrating the PC or surrounding renal parenchyma, regardless of whether the infiltration was partial or total. PC absence and PC invasion were defined as incomplete PC in our study. This retrospective study was approved by the institutional review board of Nanjing Drum Tower Hospital. Due to the retrospective nature of the study, the informed consent was waived.

### 2.1 Statistical analysis

Statistical analyses were performed using SPSS 23.0 software. Normality of the data was tested by Kolmogorov–Smirnov test. The difference among groups were compared using one‐way ANOVAs or chi‐square tests. For univariate and multivariate analysis, the logistic regression model was performed. A *p* value less than 0.05 was considered statistically significant.

## 3 RESULTS

A total of 147 small RCCs, including 117 clear cell RCCs (CCRCCs, 80%), 18 papillary RCCs (PRCCs, 12%), and 12 chromophobe RCCs (ChRCCs, 8%) were enrolled in the study. All small RCCs had pathologically confirmed negative surgical margins. Of note, 144 (98%) asymptomatic small RCCs were detected during the physical examination or incidental detection. The diameter of small RCCs ranged from 0.8 to 4 cm, with mean diameter of 2.87 ± 0.8 cm. Necrotic or cystic components, homogeneity, hyperenhancement pattern, and clear boundary on CT scan occurred in 26 (18%), 99 (67%), 119 (81%), and 74 (51%) tumors, respectively. The PC was complete in 87 (59%) small RCCs, while PC absence and PC invasion were found in 20 (14%) and 40 (27%) small RCCs, respectively. Other detailed imaging and clinicopathological features data are summarized in Table 1.

**TABLE 1:** Clinicopathological and computed tomography imaging features

Variable	Category	Total, *n*% (*n* = 147)
Age (years)		56.41 ± 12.4
Gender	Male	106 (72%)
	Female	41 (28%)
BMI (kg/m^2^)		23.48 ± 3.04
Side	Left	67 (46%)
	Right	80 (54%)
Hypertension	Yes	66 (45%)
	No	81 (55%)
Smoking history	Yes	22 (15%)
	No	125 (85%)
Clinical symptom	Yes	3 (2%)
	No	144 (98%)
Diameter (cm)		2.87 ± 0.8
Necrosis or cyst	Yes	26 (18%)
	No	121 (82%)
Homogeneity	Yes	99 (67%)
	No	48 (33%)
Enhancement pattern	Hyperenhancement	119 (81%)
	Hypoenhancement	28 (19%)
Boundary	Clear	74 (51%)
	Unclear	73 (49%)
Histological subtype	CCRCC	117 (80%)
	PRCC	18 (12%)
	ChRCC	12 (8%)
Nuclear grade	1–2	114 (84%)
	3–4	21 (16%)
PC status	Complete	87 (59%)
	Absence	20 (14%)
	Invasion	40 (27%)

Abbreviations: BMI, body mass index; CCRCC, clear cell renal cell carcinoma; ChRCC, chromophobe renal cell carcinoma; PC, pseudocapsule; PRCC, papillary renal cell carcinoma.

**FIGURE 1:**
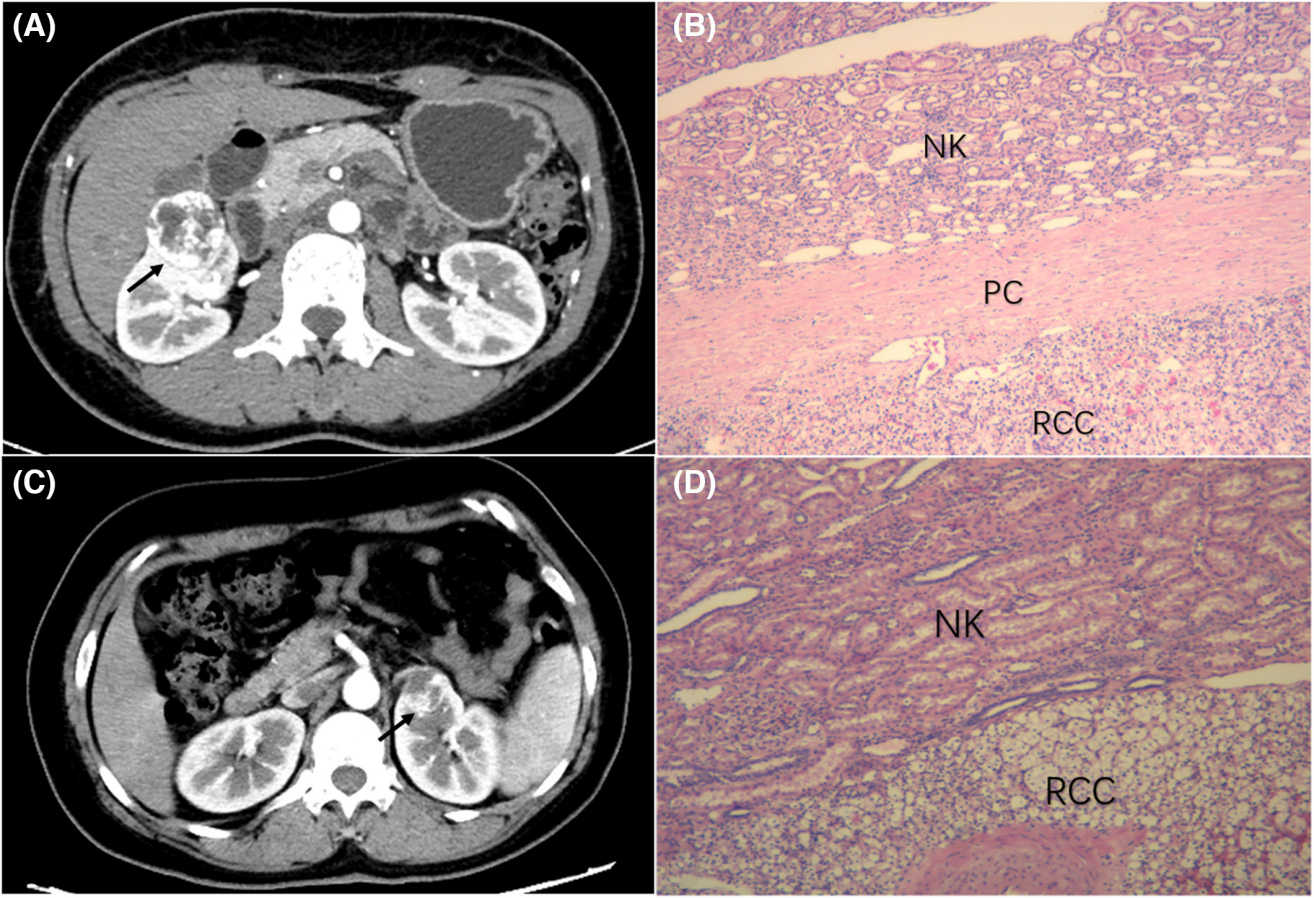
The typical computed tomography imaging feature and PC status in the clear cell renal cell carcinoma (A–D). Clear boundary (arrows) is observed at the tumor‐parenchyma interface on the corticomedullary phases image (A). Pseudocapsule is completely intact at the tumor‐parenchyma interface (B). Unclear boundary (arrows) is observed at the tumor‐parenchyma interface on the corticomedullary phases image (C). Pseudocapsule is absent at the tumor‐parenchyma interface (D). NK, normal kidney; PC, pseudocapsule; RCC, renal cell carcinoma

**FIGURE 2:**
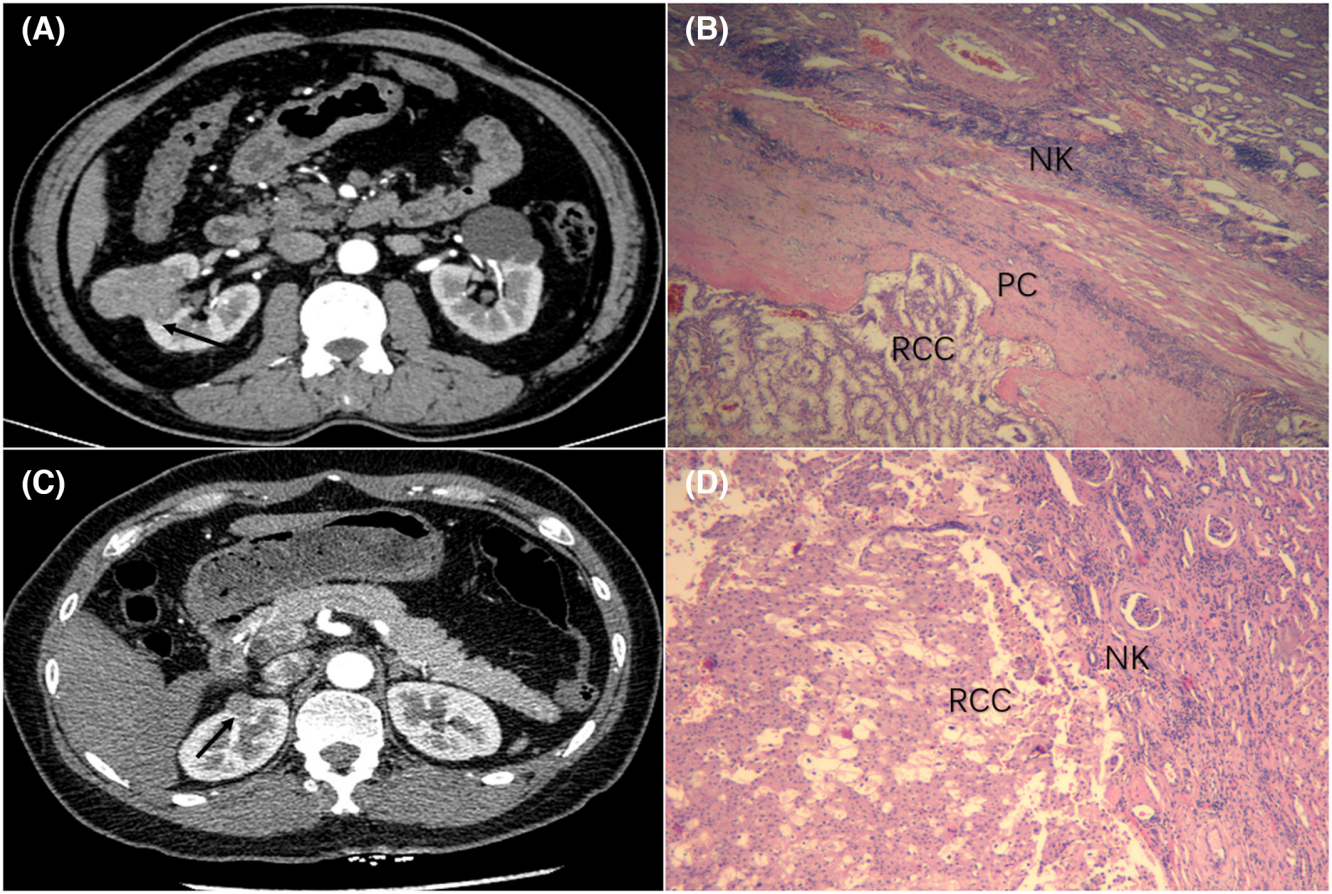
The typical computed tomography imaging feature and PC status in the papillary renal cell carcinoma and chromophobe renal cell carcinoma, respectively (A–D). The irregular tumor shows the hypoenhancement pattern and unclear boundary (arrows) on the corticomedullary phases image (A). Infiltrative tumor cells invade the surrounding pseudocapsule (B). The small tumor shows the hypoenhancement pattern and unclear boundary (arrows) on the corticomedullary phases image (C). The tumor pseudocapsule is completely disappeared at the tumor‐parenchyma interface. NK, normal kidney; RCC, renal cell carcinoma; PC, pseudocapsule

Tumor characteristics were divided based on PC status and are shown in Table 2. The proportion of female patients was highest in the PC absence group (50%) compared with the complete PC group (29%) and PC invasion group (15%). Subgroup analyses showed that the mean diameter of small RCCs differed among the groups (*p* < 0.001). Tumor diameter in the PC absence group (2.24 ± 0.93 cm) was smaller compared with that of the complete PC group (2.88 ± 0.76 cm), whereas tumors in the PC invasion group had the largest diameters (3.16 ± 0.64 cm). Of cases of PC invasion 28% were high nuclear grade. The proportion of high nuclear grade was only 7% in the PC absence group, although the difference was not statistically significant (*p* = 0.054). Compared with the other groups, the complete group showed a hyperenhancement pattern (92%) and clear boundary (63%) on a CT scan. Patients in the PRCC and ChRCC groups were combined to form the non‐CCRCC group. The difference in PC status between the two groups was statistically significant (*p* < 0.001), with 68% of patients in the CCRCC group having complete PC compared with 23% in the non‐CCRCC group. Specifically, the number of cases in the PRCC group with PC invasion was 13 (72%). Of the 12 ChRCCs, PC absence was present in 5 (42%) cases. Representative radiographic and pathologic images of PC status were shown in Figures 1 and 2.

**TABLE 2:** Tumor features were divided based on pseudocapsule status

	Pseudocapsule status			*p*
	Complete (*n* = 87)	Absence (*n* = 20)	Invasion (*n* = 40)	
Age (years, mean ± SD)	56.48 ± 12.49	56.4 ± 12.64	56.28 ± 12.40	0.996
Gender				
Male	62	10	34	**0.017**
Female	25	10	6	
BMI (kg/m^2^)	23.27 ± 3.08	23.41 ± 2.49	23.76 ± 3.25	0.788
Side				
Left	38	13	16	0.144
Right	49	7	24	
Hypertension				
Yes	42	10	14	0.334
No	45	10	26	
Smoking history				
Yes	11	4	7	0.633
No	76	16	34	
Clinical symptom				
Yes	0	1	2	0.109
No	87	19	38	
Diameter (cm, mean ± SD)	2.88 ± 0.76	2.24 ± 0.93	3.16 ± 0.64	**<0.001**
Necrosis or cyst				
Yes	16	4	6	0.86
No	71	16	34	
Homogeneity				
Yes	59	15	25	0.616
No	28	5	15	
Enhancement pattern				
Hyper‐enhancement	80	13	26	**<0.001**
Hypo‐enhancement	7	7	14	
Boundary				
Clear	55	4	15	**<0.001**
Unclear	32	16	25	
Histological subtype				
CCRCC	80	14	23	**<0.001**
Non‐CCRCC (PRCC+ChRCC)	7 (4 + 3)	6 (1 + 5)	17 (13 + 4)	
Nuclear grade				
Low	74	14	26	0.054
High	10	1	10	

Abbreviations: BMI, body mass index; CCRCC, clear cell renal cell carcinoma; ChRCC, chromophobe renal cell carcinoma; PRCC, papillary renal cell carcinoma.

Univariate and multivariate analyses were conducted to investigate factors associated with incomplete PC (Table 3). Univariable analyses revealed that hypoenhancement pattern (*p* < 0.001), unclear boundary (*p* < 0.001) and non‐ccRCC (*p* < 0.001) were related to incomplete PC. We further conducted a multivariable logistic analysis to explore the factors influencing incomplete PC. Hypoenhancement pattern (odds ratio [OR]: 4.004; 95% confidence interval [CI]: 1.07–14.987), unclear boundary (OR: 5.157; 95% CI: 2.186–12.163) and non‐CCRCC subtype (OR: 6.904; 95% CI: 2.087–22.841) were found to be independent risk factors of incomplete PC.

**TABLE 3:** Logistic regression analyses for predictors of incomplete pseudocapsule

Variables	Univariate analysis		Multivariate analysis	
	OR (95% CI)	*p*	OR (95% CI)	*p*
Age (year)	0.999 (0.973–1.026)	0.936		
Gender (male vs. female)	1.109 (0.531–2.317)	0.783		
BMI (kg/m^2^)	1.031 (0.925–1.150)	0.58		
Side (left vs. right)	1.206 (0.623–2.334)	0.578		
Hypertension (yes vs. no)	0.714 (0.367–1.39)	0.322		
Smoking history (yes vs. no)	1.551 (0.625–3.852)	0.344		
Diameter (cm)	0.972 (0.644–1.467)	0.892		
Necrosis or cyst (yes vs. no)	0.888 (0.372–2.116)	0.788		
Homogeneity (yes vs. no)	1.917 (0.909–4.043)	0.088		
Enhancement pattern (hyperenhancement vs. hypoenhancement)	11.17 (3.592–34.76)	<0.001	4.004 (1.07–14.987)	0.039
Boundary (clear vs. unclear)	3.709 (1.847–7.446)	<0.001	5.157 (2.186–12.163)	<0.001
Histological subtype (CCRCC vs. non‐CCRCC)	7.104 (2.79–18.032)	<0.001	6.904 (2.087–22.841)	0.002
Nuclear grade (low grade vs. high grade)	1.727 (0.706–4.225)	0.231		

Abbreviations: BMI, body mass index; CCRCC, clear cell renal cell carcinoma.

## 4 DISCUSSION

This is the first study to investigate the PC status in small RCCs after TE. Complete PC was observed in CCRCC, whereas PC absence and PC invasion were common in ChRCC and PRCC, respectively. Further analysis showed that PC absence was related to small size, female, and low nuclear grade tumors, whereas PC invasion was associated with large size, male, and high nuclear grade. Furthermore, hypoenhancement pattern, unclear boundary, and non‐CCRCC subtype were found to be independent risk factors of incomplete PC.

The peritumoral PC in RCC was first reported by Cahill^17^ in 1948, and is now considered as an important tissue structure that prevents positive surgical margin status during TE. In 1984, Rosenthal et al. first reported varying degrees of PC invasion at the tumor‐parenchyma interface.^18^ Minervini et al. proposed that the probability of PC penetration was greater on the parenchymal side compared with the perirenal fat tissue side.^12^ Considering the effect of different measurement sites on the PC status, we evaluated PC status on the parenchyma side in this study. In previous studies, the definition of PC status was vague. Takagi et al. only investigated the absence of PC in Stage T1 RCCs, ignoring the influence of PC invasion.^9^ In a study on renal tumor capsule invasion scoring system, the impact of PC absence was ignored,^3^ despite PC absence being common in small RCCs. Based on findings from the previous studies, we successfully divided PC status at the tumor‐parenchyma interface into three groups as follows: complete PC, PC absence, and PC invasion.

The presence of peritumoral PC is considered to be a hallmark of malignant renal tumors because it is rare in benign tumors.^5^ Among 147 small RCCs in our study, 14% (20/147)of tumors showed the absence of peritumoral PC. According to previous studies, PC was present in 89%–92% of CCRCC, 35%–93% of PRCC, and 30%–53% of ChRCC,^5^, ^9^, ^14^ while the proportion in our study was 88%, 94%, and 58%, respectively. ChRCC is associated with relatively good prognosis, and a high proportion of PC absence.^3^, ^9^ In our study, PC was absent in 5 (42%) out of 12 ChRCCs, a proportion that was slightly lower than the previously reported (47%–70%).^5^, ^9^, ^14^ The risk of PC absence was significantly greater when the tumor diameter was less than 34 mm in stage T1 RCC.^9^ In our study, the tumor diameter of the PC absence group was significantly smaller (2.24 ± 0.93) compared with the complete PC (2.88 ± 0.76) and PC invasion (3.16 ± 0.64) groups. Interestingly, lower nuclear grade (14/15, 93%) was more frequent in the PC absence group, although the difference was not statistically significant. Moreover, female patients in our study were associated with PC absence, which is consistent with findings from a previous study.^8^ The reason for the difference remains unclear and requires further study.

PC invasion was previously reported in 68% and 76% of cases,^3^, ^8^ which was higher than the proportion (27%) reported in our study. The discrepancy in the results could be due to the inclusion of patients with small RCC only in our study. A previous study reported that PC invasion was found in 56% of PRCCs after in vitro TE on radical nephrectomy specimen.^4^ In the present study, the proportion of PC invasion was 70% after in vivo TE. Several studies have demonstrated that PC invasion is associated with large and high nuclear‐stage tumors.^4^, ^9^, ^19^ Each 1 cm increase in tumor diameter was correlated with a 41% increase in the risk of PC invasion.^12^ Compared with the other two groups, the PC invasion group had the largest tumor size in our study. The proportion of high nuclear grade in our study was higher in the PC invasion group (28%) than in the complete PC (12%) and absence (7%) groups, although the difference was not statistically significant (*p* = 0.054).

PC absence and invasion are important factors influencing the surgical options and prognosis of RCC patients.^4^, ^8^, ^9^, ^20^, ^21^ In this study, PC absence and PC invasion were defined as incomplete PC. PC is composed of collagen fibers and smooth muscle bundles and is formed by the pressure exerted by the proliferating renal tumor cells on the adjacent normal renal parenchyma.^5^ Previous studies reported that CCRCC was associated with tougher and thicker PC composed of higher collagen fiber density compared with PC in non‐CCRCC.^6^, ^21^ This may explain why some surgeons hold the view that TE is not suitable for non‐CCRCC patients.^9^, ^14^, ^21^ Peritumoral PC is the natural surgical dissection plane for TE. Incomplete PC may lead to the accidental tumor incision during the TE and consequently to a positive surgical margin.^22^ Several studies have indicated that a positive surgical margin is associated with a poor prognosis.^9^, ^23^, ^24^ Although positive surgical margin was not observed in our TE specimen, several small RCCs with incomplete PC showed that the tumor cells were close to the surgical margin (<1 mm). TE surgery should be carefully taken into consideration for small RCCs with incomplete PC.

The relationship between prognosis and incomplete PC has been widely investigated in recent years. A study by Xi et al. involving 1307 patients found that incomplete PC resulted in clinically significant adverse effects on the prognosis of patients.^8^ In our study, large size, male, and high nuclear grade were frequently seen in small RCCs with PC invasion. These tumor features were reported to be associated with poor prognosis. In addition, we found that PC absence was related to small size, female, and low nuclear grade tumors, which appeared to result in good prognosis.^25^, ^26^, ^27^ This is inconsistent with previous findings probably due to differences in tumor stage of patients in our study in relation to patients in other studies. Moreover, we only included patients with small RCCs instead of all renal tumors as reported in other studies. Xi et al. reported that high nuclear grade was present in 44% of tumors with PC absence, while high nuclear grade was only present in 7% of tumors with PC absence in our study.^8^ Although PC absence in small RCCs may not correspond to poor prognosis, PC absence increase the risk of positive surgical margin during TE. Therefore, TE should be used with caution in tumors with incomplete PC.

At present, most studies about peritumoral PC focused on postoperative pathological features.^4^, ^5^, ^6^, ^11^, ^12^, ^21^ Few studies have concentrated on the relationship between preoperative imaging features and PC status. Several studies have demonstrated that CT and magnetic resonance imaging (MRI) can accurately detect PC presence.^10^, ^14^, ^28^ Since all our cases were early‐stage renal tumors, MRI was not used for imaging since it is not a routine preoperative examination. Therefore, multiple CT imaging features were used to evaluate the PC status in this study. Although several studies have reported a relationship between PC status and tumor necrosis,^7^, ^29^ necrosis was not able to predict PC status in our study. As a matter of fact, it was difficult to distinguish tumor necrosis from cystic lesions using CT scan. Univariate and multivariate analyses showed that hypoenhancement patterns and unclear boundaries were independent risk factors of incomplete PC in our study. Therefore, TE should be used with caution in tumors with such imaging features.

There are several limitations in this single‐center retrospective study. First, a small sample size was used which limits the generalizability and accuracy of our conclusions, especially the small numbers of patients with PRCC and ChRCC. Second, PC status has not been clearly defined in previous studies. Thus, we categorized PC status at the tumor‐parenchyma interface into three distinct subtypes: complete PC, PC absence, and PC invasion and evaluated the factors influencing PC status. Third, we did not investigate the association between PC status and prognosis due to the short follow‐up period.

## 5 CONCLUSIONS

This study found that hypoenhancement pattern, unclear boundary, and non‐clear cell RCC subtype were independent risk factors of incomplete PC in small RCCs. Because incomplete PC increases the risk of positive surgical margin during TE and is associated with poor prognosis, tumors with incomplete PC should be closely monitored during preoperative surgical planning or postoperative follow‐up.

## AUTHOR CONTRIBUTIONS

Xiang Dong and Sheng Pan contributed equally to this research. Xiang Dong: conception, writing, editing. Sheng Pan: Data collection, assembly of the data. Xiaodie Zhou: Reviewed pathologic specimens. Wenliang Ma: Data analysis, review. Hongqian Guo: Conception, design. Weidong Gan: design, supervision, review. All authors approved the final manuscript.

## CONFLICT OF INTEREST

The authors confirm that there are no conflicts of interest.

## ETHICS APPROVAL STATEMENTS

The protocol for this research project has been approved by the institutional review board of Nanjing Drum Tower Hospital and it conforms to the provisions of the Declaration of Helsinki. Due to the retrospective nature of the study, the informed consent was waived.

## Data Availability

The data that support the findings of this study are available from the corresponding author upon reasonable request.
